# Supplementary figures and images for: Neural correlates of appetitive extinction learning: an fMRI study with actively participating pigeons
Source: Sci Rep. 2026 May 27;16:16455. doi: 10.1038/s41598-026-54678-8 (PMC13216286; doi:10.1038/s41598-026-54678-8)

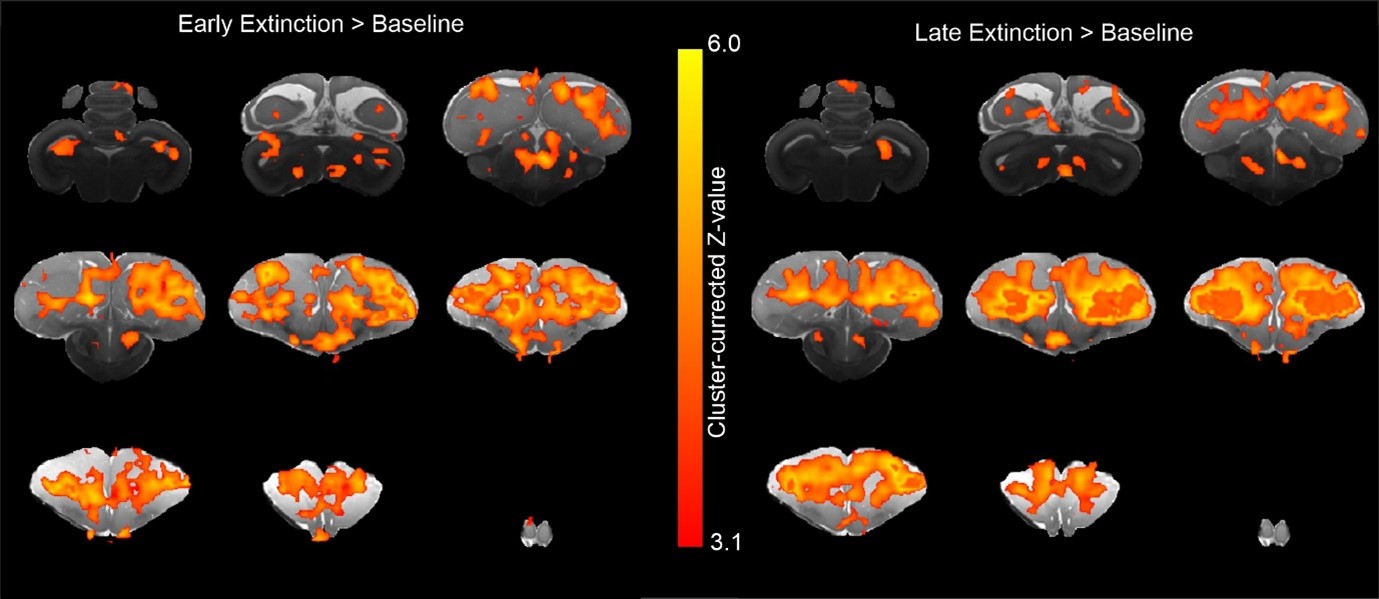

Supplement: Supplementary file 1 — Supplementary Material 1 [file 41598_2026_54678_MOESM1_ESM.jpg]
